# Self-reported pain among Cambodian Americans with depression: patient-provider communication as an overlooked social determinant

**DOI:** 10.1186/s41687-022-00504-4

**Published:** 2022-09-23

**Authors:** S. Megan Berthold, Richard Feinn, Angela Bermudez-Millan, Thomas Buckley, Orfeu M. Buxton, Sengly Kong, Theanvy Kuoch, Mary Scully, Tu Anh Ngo, Julie Wagner

**Affiliations:** 1 Division of Behavioral Sciences and Community Health, University of Connecticut School of Dental Medicine, Farmington, USA; 2 Department of Public Health Sciences, University of Connecticut School of Medicine, Farmington, USA; 3 University of Connecticut School of Social Work, Hartford, USA; 4 Department of Pharmacy Practice, University of Connecticut School of Pharmacy, Storrs, USA; 5 Department of Biobehavioral Health, Pennsylvania State University, State College, USA; 6 Department of Medical Sciences, Frank H. Netter School of Medicine, Quinnipiac University, Hamden, USA; 7 Khmer Health Advocates, West Hartford, USA; 8 Edith Nourse Rogers Memorial VAMC, Bedford, USA; 9 Department of Psychiatry, University of Connecticut School of Dental Medicine, Farmington, USA

## Abstract

**Objectives:**

Pain is common among torture survivors and refugees. Clear communication about one’s pain is vital to timely and precise diagnosis and treatment but is rarely recognized as a social determinant of health. We examined whether self-reported difficulty communicating with their health care provider, along with standard social determinants, is associated with self-reported pain in Cambodian American refugees.

**Methods:**

Secondary data analysis was conducted on *n* = 186 baseline assessments from a diabetes prevention trial of Cambodian Americans with depression. Bilingual, bicultural community health workers (CHWs) conducted surveys including social determinants of health and past week pain occurrence and interference.

**Results:**

The sample was 78% female, modal household income = $25,000, mean age = 55 years, and mean education = 6.9 years. About one-third had private insurance and two-thirds could not speak English conversationally. The average pain score was 2.8 on a scale from 0–8 with 37% reporting no pain at all. In bivariate analyses, predictors of higher pain scores were higher difficulty understanding healthcare provider, depressive symptoms, trauma symptoms, food insecurity, and social isolation; predictors of lower pain scores were higher years of education, income, English language proficiency, social support, working, and having private insurance. In the multivariate backward elimination model only two predictors were retained: difficulty understanding healthcare provider and depressive symptoms.

**Discussion:**

We propose that healthcare communication is a modifiable social determinant of health. Healthcare institutions should receive the resources necessary to secure patients’ rights to clear communication including trained community health workers.

## Plain English summary

Pain is a common health problem among torture survivors and refugees. Clear communication about one’s pain supports timely and precise diagnosis and treatment. The relationship between communication with health providers and pain is understudied. We examined whether Cambodian American refugees’ reports of difficulty communicating with their health care provider was associated with their reports of pain. We analyzed data collected by Cambodian community health workers from a sample of 186 Cambodian American adults with depression who did not have diabetes. More than three quarters were female with an average age of 55 years and an average of almost seven years of education. About one-third had private insurance and two-thirds could not speak English conversationally. A little more than a third reported no pain at all. There were multiple factors associated with higher reported pain scores including: greater difficulty understanding healthcare provider, depressive symptoms, trauma symptoms, food insecurity, and social isolation. Those who reported lower pain scores had more education, higher income, spoke English more fluently, had more social support, were working, and had private insurance. When we analyzed the influence of all of these factors together, only difficulty understanding one’s healthcare provider and depressive symptoms predicted level of pain. Difficulty communicating with the healthcare provider is a factor that can be addressed with intervention to improve health and reduce pain. Healthcare institutions should be supported with necessary resources, such as trained community health workers, to secure patients’ rights to be able to communicate with their health providers.

## Introduction

Health outcomes are influenced considerably by non-medical social factors. These social determinants of health are “conditions or circumstances in which people are born, grow, live, work, and age. These conditions are shaped by political, social, and economic forces” [1]. The literature documents a long and growing list of social determinants of health. Among those most predominant are income, education, stress, early life experiences, social exclusion, social support, work, unemployment, addiction, food, and transportation [2]. The importance of healthcare system factors has also been recognized. Patient-provider communication challenges are an often overlooked yet addressable healthcare system factor especially for Limited English Proficient (LEP) patients [3].

Pain is a commonly reported health problem in torture survivors and refugees [4], and Cambodian genocide survivors are among those with an expected high prevalence of pain. Ineffective communication regarding pain due to language barriers and cultural differences have been described by Primary Care Providers as key barriers to treating and providing culturally congruent care to LEP Hmong patients with pain [5].

Language barriers with health providers are common among Cambodian American genocide survivors and are among the greatest barriers to their accessing healthcare [6]. Healthcare providers see clear and nuanced communication about one’s pain as vital to timely and precise diagnosis and treatment [7]. LEP patients struggle in communicating with health providers about their pain [5] and patients have reported that communication problems led to increased anxiety, frustration about providers not believing their reports of the severity of their pain and its impact, and disengagement from diagnosis and treatment as a result [8].

Ease of patient-provider communication may be influenced by the provider’s level of language proficiency and/or the patient’s proficiency, with individual variation in the relative contribution of each. But ultimately the healthcare system must implement strategies to adequately facilitate patient-provider communication. It is in this sense that difficulty communicating is a healthcare system factor that we consider to be a social determinant of health.

We examined whether self-reported difficulty communicating with their health care provider predicted self-reported pain in Cambodian American refugees beyond the contribution of other commonly reported social determinants.

## Methods

### Design

Secondary descriptive data analysis was conducted on baseline assessments from a diabetes prevention trial for Cambodian Americans with depression, clinicaltrials.gov identifier NCT02502929. Details of the methods have been reported previously [9]. Baseline data were collected from March 2016 to March 2019 on a rolling basis as enrollment in the trial continued.

### Participants and sampling

All participants with baseline data were included in these analyses. The *n* = 186 resulted from a power analysis of the primary outcome of the trial (depression and HbA1c) [9]. Inclusion criteria were: (1) Cambodian or Cambodian-American; (2) Khmer speaking; (3) aged 35–75; (4) currently living in Connecticut, Rhode Island or Massachusetts (northeastern U.S.); (5) lived in Cambodia during 1975–1979 (the Pol Pot regime); (6) ambulatory; (7) take meals by mouth; and (8) elevated risk for diabetes per the American Diabetes Association Risk Test. Participants were also required to meet criteria for depression by (a) current antidepressant medication, and/or, (b) elevated depressive symptoms on the Khmer language Hopkins Symptom Checklist [10]. Exclusion criteria were: extant type 2 diabetes; seeing or hearing problems that would interfere with group sessions; major medical problems requiring intensive treatment; pregnancy or planning pregnancy; serious thinking or memory problems (e.g., schizophrenia or dementia); and 3 or more days in a psychiatric hospital or self-harm in the past 2 years.

### Procedures

The study was conducted in accord with the principles of the Declaration of Helsinki and approved by the UConn Heaith institutional review board. Participants signed informed consent forms in their preferred language (Khmer or English). Bilingual, bicultural community health workers (CHWs) conducted surveys. Participants were paid $10 each in gift cards to a local pharmacy for completing the surveys and HbA1c assessment.

### Measures

#### Demographic and clinical characteristics

Participants reported their age and sex. They also reported their ability to speak and read English, each on a 4-point scale from 0 = “not at all” to 4 = “very well”. HbA1c is the gold standard measure of glycemia. HbA1c was assayed at Quest laboratory and values are reported in percent units according to the National Glycohemoglobin Standardization Program (NGSP).

#### Social determinants

##### Healthcare system factors

Participants were asked how often in the past year they experienced difficulty communicating with their healthcare provider because of a language difference using a 5-point scale from “never” to “always” with higher scores indicating greater difficulty. They were asked a similar question referencing their pharmacist. Participants reported insurance status (Medicare, Medicaid vs private).

##### Educational attainment

Participants were asked, “How many years of education do you have?”.

##### Early adverse experiences

Participants were asked to report the number of years they had lived in the Khmer Rouge regime, and how many years they had lived in a refugee camp.

##### Income

Given the generally low household income of the population, income was categorized as < $20,000, $20,000-$30,000, $31,000-$40,000 or > $40,000.

##### Work

Participants were asked to choose their employment status: full time, part time, retired, homemaker, disabled, unemployed looking for work, or other.

##### Food

Food insecurity was assessed using the 6-item U.S. Household Food Security Survey (HFSS) module [11] using a 3-month time reference. The sum of affirmative responses produces a scale score (0–6). Higher scores indicate greater food insecurity.

##### Transportation

We asked two questions including, “can you drive?” and “do you have access to a car?” Responses options were 0 = ”no” and 1 = ”yes”; they were summed for a transportation score from 0–2.

##### Stress

We measured symptoms of depression with the 15-item depression subscale of the Khmer language version of the Hopkins Symptom Checklist [10]. We assessed symptoms of Post-Traumatic Stress Disorder (PTSD) with the 16-item symptom subscale of the Khmer language version of the Harvard Trauma Questionnaire [10].

##### Social support

Participants responded to four items from the Patient-Reported Outcomes Measurement Information System (PROMIS) test bank [12] and one item from the Enriched Social Support Instrument [13]. Response options were on a 5-point scale with higher scores indicating greater social support.

##### Social isolation

One item from PROMIS test bank [12] asks about “feeling isolated from other people”. Response options were on a 5-point scale with higher scores indicating greater isolation.

##### Addiction/substance use

Participants who endorsed drinking alcohol in the past year [14] went on to answer the four-item substance use subscale of the COPE [15] which assesses drinking alcohol as strategy to cope with distress. Response options are on a four-point scale from “I usually don’t do this at all” to “I usually do this a lot”.

#### Patient reported outcome variable

Pain. We measured past week pain occurrence and interference. Participants were asked, “Did you experience pain in the past 7 days?” Response options were “yes = 1” or “no = 0”. Participants who responded “yes” were asked, “Did pain make it difficult for you to do your day-to-day activities?” Participants who responded “yes” were asked, “How difficult did pain make it to complete your day-to-day activities?” Response options were “a little bit difficult = 1”, “moderately difficult = 2” or “very difficult = 3”. Two questions from the Pittsburg Sleep Quality Index (PSQI) [16] asked: “During the past month, have you had trouble sleeping because you have physical pain?” (yes = 1 or no = 0); affirmative responses were followed up with “How often?” with response options ranging from rarely = 1 to very frequently = 3. All responses were summed and pain scores could range from 0 (no pain) to 8 (pain that makes daily activities very difficult and with frequent trouble sleeping).

### Statistical analysis

The pain score ranged from 0 to 8 with excess zeros (37%) and so a generalized linear model with negative binomial distribution and logit link was used to model predictors of pain. Bivariate analyses were performed for each predictor separately followed by a backward multivariate model that started with all significant bivariate predictors and removed non-significant predictors one at a time until only significant predictors remained. The analyses were conducted in SPSS v27.

## Results

The *n* = 186 participants (Table 1) were 87% female, mean = 55 (SD = 8.6) years old, with median household income = $25,000 and years of education mean = 6.9 (SD = 5.0). One-third (33%) had private insurance and 30% could speak English conversationally. Because participants were excluded for diabetes, average HbA1c was in the normal range (mean = 5.5%, SD = 0.4). HbA1c was not associated with pain scores.

**Table 1:** Patient Characteristics (*n* = 188)

Characteristic	Frequency	Percentage
*Gender*		
Female	145	78.0%
*Age*		
Mean ± SD	55.2 ± 8.9	
*Health Insurance*		
Medicare/Medicaid Private Other Don’t Know/Refused	106 64 8 8	57.0% 34.4% 4.3% 4.3%
*Household Income*		
< $20,000 $20,000—$30,000 $31,000—$40,000 Over $40,000	81 41 17 27	48.8% 24.7% 10.2% 16.3%
*Working*		
Yes	67	36.0%
*Education Years *		
Mean ± SD	6.9 ± 5.0	
*English (% conversational/very well)*		
How well do you speak? How well do you read?	59 55	31.7% 29.6%
*Years in Khmer Rouge*		
Mean ± SD	3.2 ± 1.1	
*Years in Refugee Camp*		
Mean ± SD	3.0 ± 5.2	
*Food Insecurity*		
Yes	39	21.3%
*Access to Car*		
Yes	139	74.7%
*Difficulty Understanding Provider*		
Sometimes/Often/Always	80	43.6%
*Difficulty Understanding Pharmacist*		
Sometimes/Often	9	5.6%
*Social Isolation*		
Sometimes/Usually/Always	70	28.0%
*Social Support*		
Mean ± SD	2.8 ± 0.9	
*Post-traumatic stress symptoms*		
Mean ± SD	1.9 ± 0.6	
*Depressive symptoms*		
Mean ± SD	1.9 ± 0.7	
*Pain Score*		
0 1–2 3–4 5–6 7–8	68 22 40 32 24	36.6% 11.8% 21.5% 17.2% 12.9%

The average pain score was 2.8 (SD = 2.7). In bivariate analyses (Table 2), predictors of higher pain scores were higher difficulty understanding healthcare provider, depressive symptoms, trauma symptoms, food insecurity, and social isolation. In bivariate analyses, predictors of lower pain scores were higher years of education, income, English language proficiency, social support, working, and having private insurance. In the multivariate backward elimination model only two predictors met criteria to be retained in the final model: difficulty understanding healthcare provider and depressive symptoms.

**Table 2:** Bivariate and Multivariate Results from Negative Binomial Model Predicting Pain Score

Predictor	Bivariate results			Multivariable results		
	Estimate	Std Err	*P*-value	Estimate	Std Err	*P*-Value
Female	.351	.212	.099			
Age	.013	.011	.244			
Years Education	− .059	.018	.001			
Income	− .202	.085	.017			
Working	− .614	.185	< .001			
Private Insurance	− .748	.190	< .001			
English Speaking Proficiency	− .250	.110	.022			
English Reading Proficiency	− .214	.090	.017			
Access to Car	− .206	.194	.290			
Drinking to Cope	.211	.121	.080			
Food Insecurity	.701	.203	< .001			
Years Khmer Rouge	.118	.077	.124			
Years in Refugee Camp	.029	.019	.127			
Difficulty Understanding Provider	.207	.063	< .001	.135	.066	.041
Difficulty Understanding Pharmacist	.564	.328	.086			
Social Support	− .299	.098	.002			
Social Isolation	.180	.059	.002			
Trauma symptoms	.977	.159	< .001			
Depressive symptoms	.945	.147	< .001	.879	.149	< .001

## Discussion

Whereas many social determinants were associated with pain, difficulty communicating with the healthcare provider and depressive symptoms were uniquely associated with greater pain beyond all other social determinants. Difficulty communicating with one’s provider is not typically considered as a social determinant [17].

We interpret our findings to mean that greater difficulty communicating is associated with greater pain because of the need for a detailed and nuanced clinical pain assessment. Communication affects every aspect including the ability of the patient to volunteer or endorse the experience of pain, describe it adequately, respond to clarifying questions, and understand recommendations for further assessment and/or treatment. If this communication is difficult, the provider may not be able to correctly diagnose and treat pain and the patient may not be able to follow up on clinical recommendations.

The degree to which Cambodian American genocide survivors are able to communicate with their health care providers is vital to their providing an accurate account of pain location, radiation, mode of onset, character, and temporal pattern. A symptom analysis includes quality, frequency, intensity, and duration of pain, aggravating and alleviating factors, associated affective and behavioral responses, and any treatments the patient has tried and respective treatment responses.

Beyond language per se, Cambodian American refugees have historical factors related to pain (e.g., torture and beatings during the Khmer Rouge genocide) which may increase distress during pain communication. Patients with a history of traumatic life events can become distressed in medical encounters especially if the encounter pertains to a medical problem linked to the trauma. For example, neurogenic pain is known as “*spuck*” in Cambodian. It is a not uncommon report that patients associate with beatings they endured in the genocide. Reporting “*spuck*” may be distressing for patients.

Our findings that higher depressive symptoms are uniquely associated with pain is consistent with findings from other populations [18] and underscores that interpersonal communication can be hindered by emotional distress [19]. The experience of pain complaints among people with major depressive disorder is common. A recent meta-analysis found that overall prevalence of physical pain symptoms in depression patients was 55.2%, with a point prevalence of 64.2% and a 12-month prevalence of 57.0% [20].

### Implications

It is noteworthy that, whereas English language speaking and reading proficiency – patient characteristics—were associated with pain, they were not retained in the final model. Rather, difficulty communicating with the provider was retained. Difficulty communicating with one’s provider is modifiable and has implications for health policy. Despite federal and some state requirements to provide qualified interpreters, many hospitals and other health facilities receiving federal funds in the United States do not [21]. Healthcare institutions should be supported with the necessary resources to secure patients’ rights to communication access. There are clear benefits to working with trained CHWs who can not only provide translation services but also interpret cultural idioms for discomfort and pain, especially those associated with traumatic events. They can also provide culturally appropriate health education and counseling in the community and advocate for patient care [22]. Our findings support policies that promote this expanded role of CHWs. Relying on casual interpreters, i.e., friends or family members of the patient, is often the default but is discouraged because it can cause both patient and provider to censor the information exchange especially when the casual translator is a child [23].

### Limitations

Results should be interpreted in light of limitations including a relatively small sample from a single U.S. region. The majority of women is explained by historical factors; more men died during the Cambodian genocide compared to women, 33.5% vs. 15.7% [24]. Women commonly experience more chronic pain than men and future studies should explore gender differences [25]. The cross-sectional design does not allow us to examine temporal precedence and it is possible that these relationships may be recursive. Our measure of pain did not include specifiers such as duration (i.e., chronic vs acute) or type (e.g., disease vs injury) or pain management strategies. Our list of social determinants was not exhaustive (e.g., we did not have a measure of housing). The use of backward elimination can sometimes result in a final model different from other selection procedures and could remove a variable with a strong correlation with the outcome if it overlaps with other predictors.

## Conclusions

The healthcare system should facilitate communication through a variety of institutional and policy changes including increasing the number of providers who speak the language of their patients, hiring interpreters and CHWs, and targeted health literacy efforts.

## Data Availability

Data available upon request to corresponding author.
